# New image‐processing technology for endoscopic ultrasound‐guided fine‐needle aspiration biopsy specimen evaluation in patients with pancreatic cancer

**DOI:** 10.1002/deo2.21

**Published:** 2021-06-09

**Authors:** Kosuke Okuwaki, Hiroshi Imaizumi, Mitsuhiro Kida, Hironori Masutani, Masafumi Watanabe, Kai Adachi, Masayoshi Tadehara, Akihiro Tamaki, Tomohisa Iwai, Hiroshi Yamauchi, Rikiya Hasegawa, Toru Kaneko, Takahiro Kurosu, Wasaburo Koizumi

**Affiliations:** ^1^ Department of Gastroenterology Kitasato University School of Medicine Kanagawa Japan

**Keywords:** endoscopic ultrasound‐guided fine‐needle aspiration, endosonography, histology

## Abstract

**Objectives:**

We evaluated the usefulness of a newly developed system with which the total amount of whitish cores in endoscopic ultrasound‐guided fine‐needle aspiration biopsy (EUS‐FNAB) samples is automatically calculated (automated multiband imaging system [AMUS]).

**Methods:**

From 30 prospectively enrolled patients suspected of having pancreatic cancer, four EUS‐FNAB specimens per patient were obtained. Following AMUS calculations, two specimens were prepared after stereomicroscopy‐guided manual division into whitish and reddish sections (isolation group), and the other two were prepared without such division (no‐isolation group). The relation of the AMUS results pertaining to the length of the manually measured whitish cores (stereo‐microscopically visible white core [SVWC]) and the sample suitability for pathologic evaluation were analyzed.

**Results:**

Histological diagnostic accuracy was 90%; median SVWC length, 14 mm; and median area of whitish core calculated using the AMUS, 13 mm^2^. The SVWC length correlated with whitish core amount (ρ = 0.83, *p* < 0.01) and adequacy score (ρ = 0.50, *p* < 0.01). The whitish core amount correlated with the adequacy score (ρ = 0.40, *p* < 0.01). The area under the receiver‐operating characteristic curve calculated for whitish core amount with respect to the histological diagnosis was 0.84 (*p* < 0.01; cutoff ≥ 8 mm^2^, sensitivity 92.5%). Subgroup analysis (isolation vs. no‐isolation group) revealed no significant between‐group differences in the median histological adequacy (*p* = 0.27) or tumor cell content ratio (*p* = 0.28). The median scores for degree of blood contamination were significantly lower in the isolation group than in the no‐isolation group (*p* < 0.01).

**Conclusion:**

AMUS is a simple on‐site verification procedure for determining the appropriate sampling tissue quantity for high diagnostic accuracy.

## INTRODUCTION

Recent meta‐analyses have shown that the sensitivity and specificity of endoscopic ultrasound‐guided fine‐needle aspiration biopsy (EUS‐FNAB) in diagnosing solid pancreatic lesions were 84%–89% and 96%–99%, respectively.^1^, ^2^, ^3^ We previously developed a procedure for sample isolation processing using stereomicroscopy (SIPS) to prepare high‐quality tissue specimens for EUS‐FNAB as an alternative to rapid on‐site evaluation (ROSE) for patients with mediastinal or upper abdominal solid masses.^4^ In SIPS, stereo‐microscopically visible white cores (SVWCs), which are useful for histological diagnosis, are separated from red components (red blood cells and fibrin). We observed a high sensitivity (91.4%) for tissue analysis using FNA for mediastinal or upper abdominal solid masses, including pancreatic neoplasms and subepithelial lesions, with an SVWC cutoff value ≥ 11 mm. Using a FNB, we achieved a similarly high sensitivity for malignant diagnosis (98.7%) at an SVWC cutoff value ≥ 4 mm in patients with upper gastrointestinal subepithelial lesions.^5^ Nevertheless, manual SIPS is a useful but slightly complicated procedure; therefore, it is desirable to create a new, more straightforward method for the objective estimation of the core tissue amount to be sampled. We developed a new image‐processing technology (automated multiband imaging system [AMUS]) to calculate the whitish core amount using EUS‐FNAB. To the best of our knowledge, this is the first attempt to evaluate the correlation between the SVWC lengths calculated manually by physicians and the whitish core amount calculated using the AMUS in EUS‐FNAB for patients with pancreatic cancer (PC) to investigate the usefulness of the AMUS.

## METHODS

### Study design

This single‐center prospective study enrolled consecutive patients who underwent EUS‐FNAB for pancreatic masses suspected to be adenocarcinomas at our hospital between January 2019 and November 2019. The inclusion criteria were age ≥ 20 years and pancreatic mass suspicious of malignancy requiring pathological diagnosis. Patients were excluded if the coagulation parameters were abnormal. The primary outcome was the correlation between the SVWC length calculated manually by physicians and the whitish core amount calculated by the AMUS. The secondary outcomes included the correlation between the SVWC length and histological adequacy score as well as between the whitish core amount and histological adequacy score, tumor content ratio in the pathological specimens, the cutoff value of the amount of whitish core for histological diagnosis, sensitivities of EUS‐FNAB using the cutoff value, histological diagnostic accuracy, and procedure‐related adverse events (AEs).

This study was conducted according to the tenets of the Declaration of Helsinki and approved by our institutional review board based on ethical, scientific, and medical validity (approval no.: B18‐167). All patients provided written informed consent before study participation. This study is registered at http://www.umin.ac.jp (UMIN000035095).

### EUS‐FNAB

EUS‐FNAB was performed without ROSE using a linear scanning video echoendoscope (GF‐UCT260; Olympus Medical Systems, Tokyo, Japan) and a 22‐gauge FNA needle (EZ Shot 3 Plus; Olympus Medical Systems) or a 22‐gauge FNB needle (Acquire; Boston Scientific Corp., Marlborough, MA, USA). The therapist chose the puncture needle. Following stylet withdrawal, 10–20 strokes were made with the needle inside the lesion using a 20‐ml syringe under negative pressure, and four needle passes were performed in all lesions. In the first two specimens (isolation group), a technician (one of the two designated endoscopists) measured the SVWC length and isolated the SVWC sample and red components according to the findings of our previous study.^4^ In the two remaining specimens (no‐isolation group), isolation was not performed.

Patients were examined twice for AEs: 3 h after EUS‐FNAB sampling and the following morning. The incidence of AEs up to 30 days after EUS‐FNAB sampling was evaluated during medical examinations in the outpatient clinic, based on established guidelines.^6^

### AMUS for EUS‐FNAB sample

**FIGURE 1:**
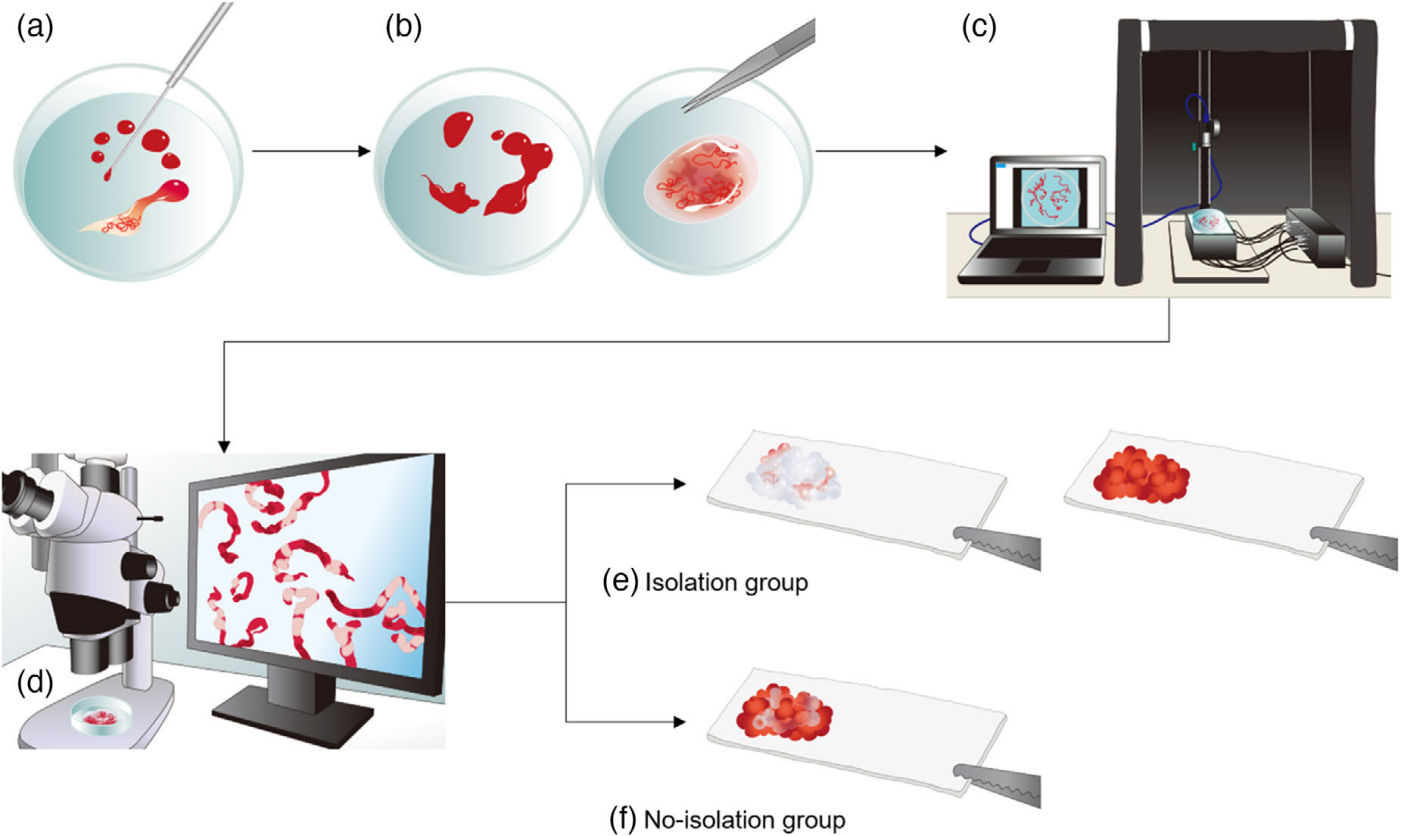
Study outflow. (a) Step 1: The sample in the puncture needle was initially extruded onto the petri dish by compressing air in the syringe and then using a stylet. (b) Step 2: The earthworm‐like core sample obtained was immersed in 10% neutral buffered formalin solution under the stereomicroscope. The liquid component remaining after extruding the sample from the needle was sent for cytologic examination. (c) Step 3: The whitish core sample was sufficiently extended onto a petri dish and soaked in 10% buffered formalin solution, irradiated using nine narrow‐band lights, and imaged to obtain multiband image data. (d) Step 4: The SVWCs were measured under the stereomicroscope (× 20–40, SZX10; Olympus Medical Systems) using a scale on the microscope monitor screen. (e) Step 5: In the isolation group, the sample in the petri dish was examined, and SVWCs and red components were dissected using injection needles. SVWCs and red components were closely aligned on separate filter papers, immersed in vessels containing 10% neutral buffered formalin, and sent for pathological analyses. (f) In the no‐isolation group, the samples were closely aligned on filter papers without isolation, immersed in vessels containing 10% neutral buffered formalin, and sent for pathological analyses. AMUS, automated multiband imaging system; SVWC, stereo‐microscopically visible white core

**FIGURE 2:**
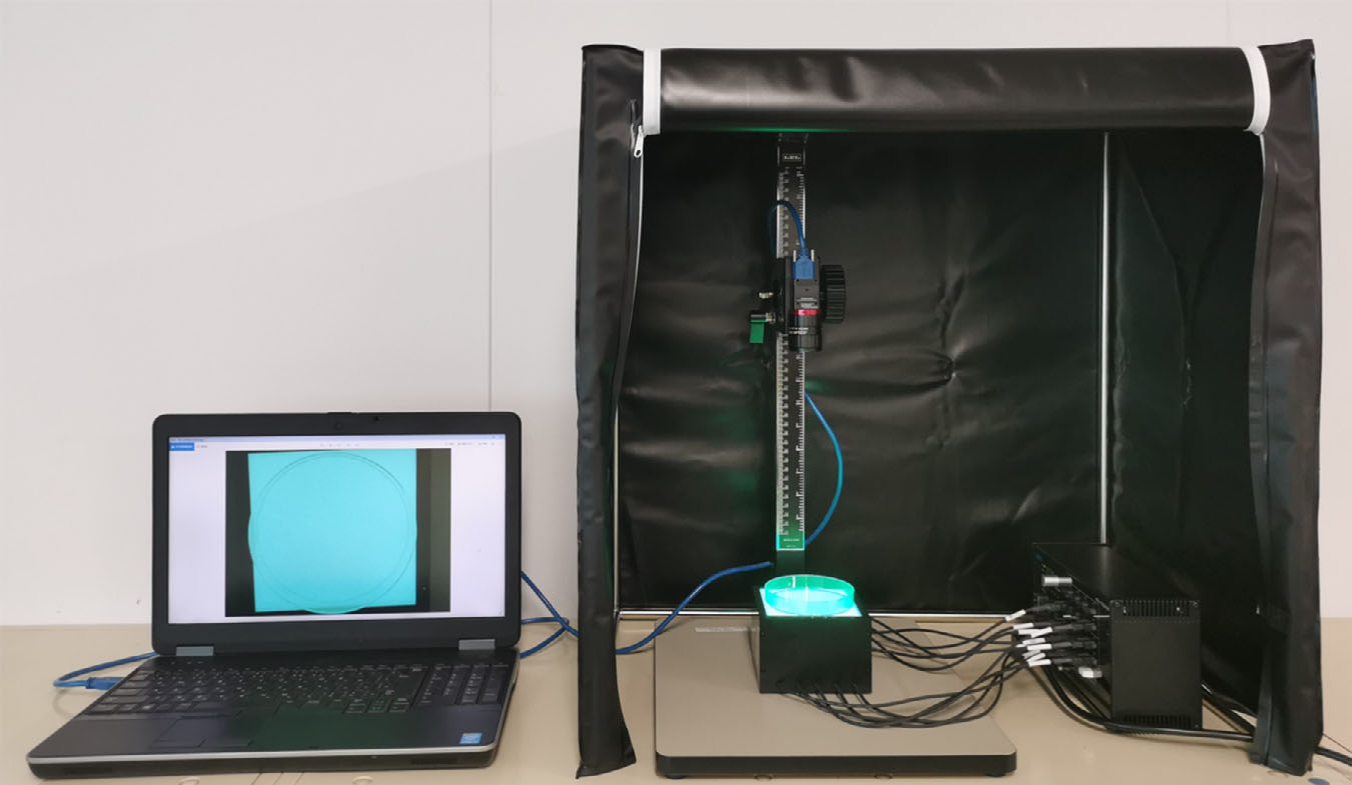
Set‐up of the automated multiband imaging device, which is a component of the AMUS. AMUS, automated multiband imaging system

The EUS‐FNAB sample was assessed using the AMUS as shown in Figure 1. The automated multiband imaging device provided by Olympus Corporation, a component of the AMUS, is shown in Figure 2. The AMUS obtained the multiband image data using nine narrow‐band lights (equipped with a multiband LED light source having peak wavelengths of 405, 430, 465, 505, 545, 600, 630, 660, and 700 nm). The whitish core regions in the multiband image were then detected using a segmentation algorithm through elimination of the influence of various concentrations of blood background by taking advantage of the property by which the spectral absorption rate of blood varies depending on the wavelength of light used to calculate the whitish core quantity (area). Specifically, the spectral transmittance of a pixel was first determined as a multidimensional vector (A). Next, the spectral transmittance of the whitish core and red component—which are the targets of segmentation—was determined as B and C, respectively. Then, the cosine similarities between A and B and A and C were determined for each pixel. The pixel was classified into the region with the highest similarity to determine whether it belonged to B or C. Finally, the area (number of pixels) of each region was calculated.

### Pathological examinations

Following formalin fixation, hematoxylin‐eosin‐stained specimens were prepared from formalin‐fixed and paraffin‐embedded tissues (FFPEs). Pathological examination was performed twice by two or more qualified pathologists. In patients who underwent surgical resection following EUS‐FNAB, the final diagnosis was considered correct if it was consistent with that of the pathological examination of the resected specimen. For patients with unresected masses, the subsequent clinical course was monitored for ≥ 6 months after EUS‐FNAB, and diagnostic imaging was performed. If the results were consistent with those of the EUS‐FNAB, then the final diagnosis was considered correct.

Two gastroenterologists (graduate students in the Department of Pathology) who were trained by an expert pathologist and were blinded to clinical information assessed each specimen to obtain the histological adequacy score, tumor cell content ratio, and the degree of blood contamination. The adequacy score was classified based on a previously reported scoring system: score 0, samples with no material; score 1, sufficient material for limited cytologic interpretation but probably representative; score 2, sufficient material for adequate cytologic interpretation but insufficient for histologic information; score 3, sufficient material for limited histologic interpretation; score 4, sufficient material for adequate histologic interpretation but a low‐quality sample (total material < 1, 10 × power field in length); and score 5, sufficient material for adequate histologic interpretation and a high‐quality sample (>1, 10 × power field in length).^7^ Specifically, using a light microscope, the percentage of tumor cell nuclei with respect to all cell nuclei in the specimen was scored (0%, score 0; 1%–19%, score 1; 20%–39%, score 2; 40%–59%, score 3; 60%–79%, score 4; and ≥80%, score 5). The degree of blood contamination was classified as follows: score 1, significant; score 2, moderate; and score 3, minimal. Two evaluators determined the classification. If the two judgments differed, then the one with the lower score was adopted.

### Statistical analyses

Power analysis could not be performed because of the study's exploratory nature; therefore, an achievable target of 30 patients was selected. Receiver‐operating characteristic (ROC) curves for the core tissue amount calculated by the AMUS concerning the histological diagnosis were plotted. The accuracy of the area under the curve (AUC) for the diagnostic yield was evaluated. The cutoff value required to obtain a histological diagnosis was calculated using the Youden index.^8^ Subgroup analyses were performed for the isolation and no‐isolation groups. Statistical comparisons were conducted using the Mann–Whitney U test and Fisher's exact probability test for categorical variables and Spearman's rank correlation coefficient for correlation. Concordance between classifications of the histological adequacy score, tumor cell content ratio, and degree of blood contamination determined by two evaluators was analyzed using the kappa coefficient. Statistical analyses were performed using R statistical packages version 3.2.4, with p‐values < 0.05 being considered statistically significant.

## RESULTS

The 30 registered subjects included 16 men (53%) and 14 women (47%), with a median age of 67 years (interquartile range [IQR]: 58–75). Overall, the median lesion's maximum diameter was 36 mm (IQR: 27–45). The lesions were located at the pancreatic head and body‐tail in 18 (60%) and 12 (40%) subjects, respectively. Four punctures were performed for each patient, and 120 samples were obtained.

Table 1 shows the EUS‐FNAB results. The three subjects for whom diagnosis could not be made based on histological findings were diagnosed following cytological examination involving an equivalent test (one patient) and histological findings via EUS‐FNAB re‐testing (two patients). All 30 patients were diagnosed with adenocarcinoma. There were no significant differences between the two groups in the histological diagnostic accuracy (*p* = 0.66).

**TABLE 1:** Results of ultrasound‐guided fine‐needle aspiration biopsy (EUS‐FNAB)

	All	Isolation group	No‐isolation group	*p*‐value
Technical success, *n* (%)	30/30 (100)			
Puncture site, *n* (%)				
Stomach	12 (40)			
D1	5 (17)			
D2	13 (43)			
Needle type, *n* (%)				
FNA needle	10 (33)			
FNB needle	20 (67)			
Accuracy of cytological diagnosis				
Per pass, *n* (%)	66/120 (55)	30/60 (50)	36/60 (60)	0.36
Per lesion, *n* (%)	20/30 (67)	17/30 (57)	19/30 (63)	0.79
Accuracy of histological diagnosis				
Per pass, *n* (%)	93/120 (78)	48/60 (80)	45/60 (75)	0.66
By SVWC sample		48/60 (80)		
By red components		24/52 (46)		
By no isolation sample			45/60 (75)	
Per lesion, *n* (%)	27/30 (90)	26/30 (87)	26/30 (87)	1.00
Overall diagnosis, *n* (%)	28/30 (93)			
Adverse events, *n* (%)	0 (0)			

The *p*‐values (isolation group vs. no‐isolation group) were determined using Fisher's exact probability test.

Abbreviations: D1, bulb of the duodenum; D2, second portion of the duodenum; FNA, fine‐needle aspiration; FNB, fine‐needle biopsy; SVWC, stereo‐microscopically visible white core.

The median SVWC length was 14 mm (IQR: 9–23 mm), measured according to the study protocol. The sensitivity to diagnose malignancy based on the histological diagnosis using the previously reported cutoff value (SVWC length ≥ 11 mm) was 99% (73/74).^4^ The sensitivity of the histological diagnosis was significantly higher for specimens with SVWC length ≥ 11 mm (99%) than those with SVWC length < 11 mm (20/46, sensitivity of 43%) (*p* < 0.01).

The 117 samples (60 from the isolation group and 57 from the no‐isolation group) were used for analysis; we excluded one sample each from three subjects as the multiband image data could not be saved due to failure of the recording medium. The median area of whitish core calculated using the AMUS was 13 mm^2^ (6–21 mm^2^). The correlations were observed between the SVWC length calculated manually by physicians and whitish core amount calculated by the AMUS (ρ = 0.83, *p* < 0.01), between the SVWC length and adequacy score (median value score 4) (ρ = 0.50, *p* < 0.01), and between the whitish core amount and adequacy score (ρ = 0.40, *p* < 0.01).

**FIGURE 3:**
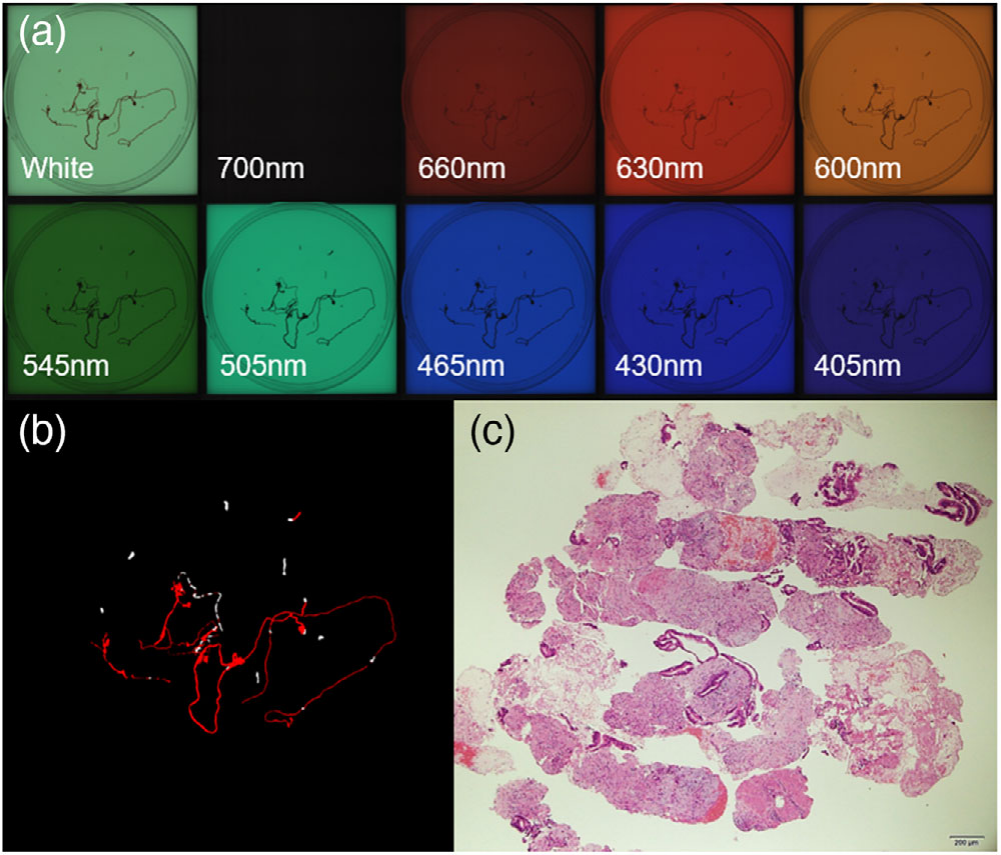
AMUS findings of a specimen of pancreatic adenocarcinoma isolated from the isolation group. (a) The multiband images obtained by the AMUS are shown. (b) The core tissue regions detected using the segmentation algorithm are shown in white. (c) Histological presentation on the bottom right image (40 × magnification). A specimen of pancreatic adenocarcinoma in the isolation group had an SVWC length of 12 mm, whitish core amount of 13 mm^2^, histological adequacy score of 5, tumor cell content ratio score of 2, and a degree of blood contamination score of 3. AMUS, automated multiband imaging system; SVWC, stereo‐microscopically visible white core

**FIGURE 4:**
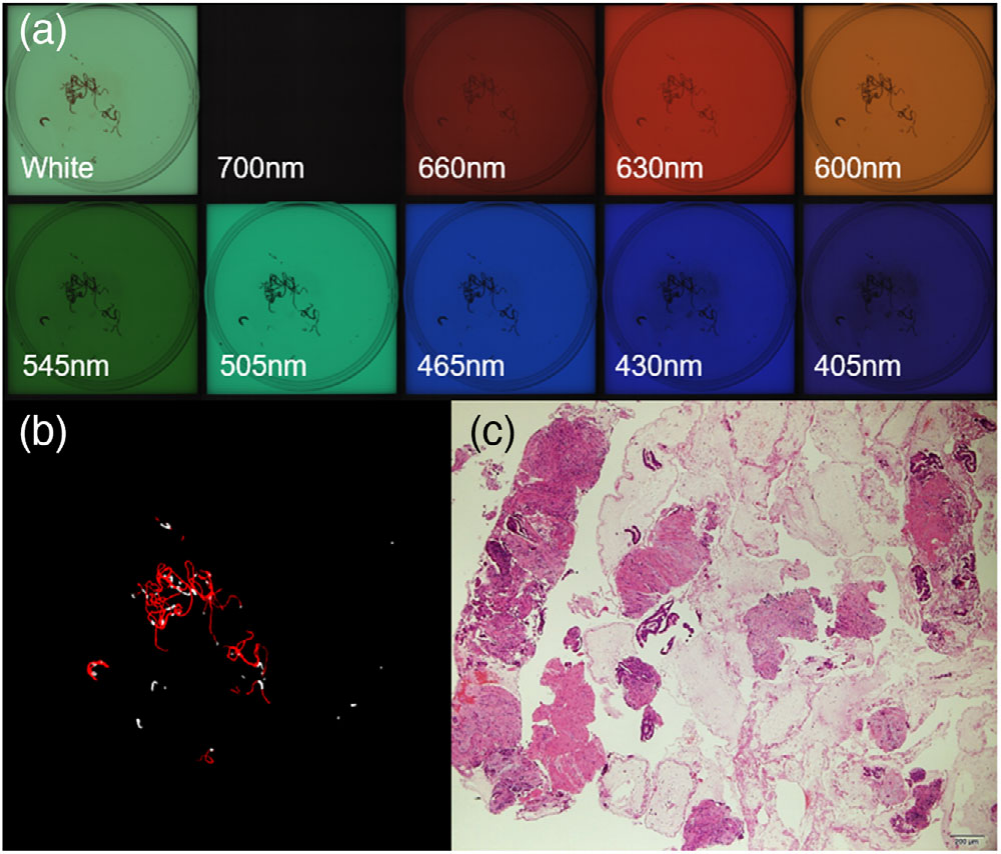
AMUS findings of a specimen of pancreatic adenocarcinoma in the no‐isolation group. (a) The multiband images obtained by the AMUS are shown. (b) The core tissue regions detected using the segmentation algorithm are shown in white. (c) The histological presentation is shown on the bottom right image (40 × magnification). The SVWC length of a specimen of pancreatic adenocarcinoma in the No‐isolation group was 12 mm, whitish core amount of 15 mm^2^, histological adequacy score of 5, tumor cell content ratio score of 2, and degree of blood contamination score of 3. AMUS, automated multiband imaging system

Table 2 shows the subgroup analysis for the isolation and no‐isolation groups. The median area of whitish core measured was not significantly different between the two groups (*p* = 0.55). The concordance rates assessed by the kappa coefficient among the evaluators for each subclassification of the pathological assessment were 0.59 (95% confidence interval [CI], 0.45–0.73; *p* < 0.01) for the histological adequacy score, 0.82 (95% CI, 0.75–0.88; *p* < 0.01) for the tumor cell content ratio score, and 0.53 (95% CI, 0.36–0.69; *p* < 0.01) for the degree of blood contamination score. There was no significant difference in the median histological adequacy (*p* = 0.27) and tumor cell content ratio (*p* = 0.27) scores. The isolation group had a significantly lower median score of the degree of blood contamination than the no‐isolation group (*p* < 0.01). Figures 3 and 4 show the multiband images obtained by the AMUS, whitish core regions detected by the segmentation algorithm, and actual histological presentations.

**TABLE 2:** Subgroup analysis between the isolation group and the no‐isolation group

	All^*^(*n* = 117)	Isolation group(*n* = 60)	No‐isolation group^*^(*n* = 57)	*p*‐value
The median SVWC length, mm, (IQR)	14 (9–24)	15 (8–23)	14 (9–24)	0.61
The median area of whitish core measured by the AMUS, mm^2^, (IQR)	13 (6–21)	12 (6–19)	14 (6–23)	0.55
The median histological adequacy score, (IQR)	4 (4–5)	5 (4–5)	4 (4–5)	0.27
The median score of the tumor cell content ratio, (IQR)	2 (1–2)	2 (1–2)	2 (1–2)	0.28
The median score of the degree of blood contamination, (IQR)	3 (2–3)	3 (3–3)	3 (2–3)	<0.01

The *p*‐values (isolation group vs. no‐isolation group) were determined using the Mann–Whitney U test.

Abbreviations: AMUS, automated multiband imaging system; IQR, interquartile range; SVWC, stereo‐microscopically visible white core.

^*^ One sample each from three subjects was excluded because of the lack of multiband image data due to failure of the recording medium.

**FIGURE 5:**
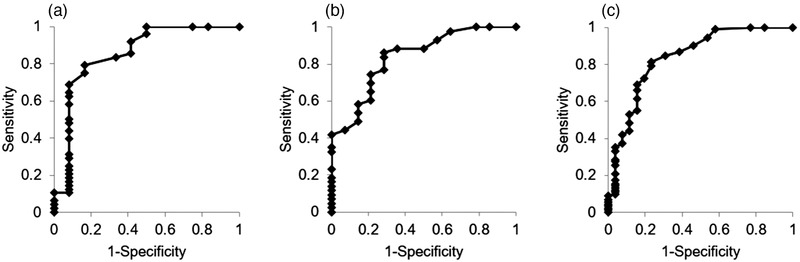
Comparison of core tissue amount measured by the AMUS concerning the histological diagnosis as determined by the ROC analysis. (a) In the isolation group, ROC curves based on the amount of whitish core measured by the AMUS concerning the histological diagnosis are shown. The AUC was 0.80 (95% CI: 0.62–0.97, *p* < 0.01). (b) In the no‐isolation group, ROC curves based on the amount of whitish core measured by the AMUS concerning the histological diagnosis are shown. The AUC was 0.84 (95% CI: 0.72–0.96, *p* < 0.01). (C) ROC curves based on the amount of whitish core measured by the AMUS concerning the histological diagnosis are shown. The AUC was 0.84 (95% CI: 0.71–0.92, *p* < 0.01). AMUS, automated multiband imaging system; AUC, area under the curve; CI, confidence interval; ROC, receiver‐operating characteristic

In the isolation group, the AUC of the ROC curves calculated for the whitish core concerning the histological diagnosis was 0.85 (95% CI: 0.70–1.00; *p* < 0.01) (Figure 5a). In the no‐isolation group, it was 0.84 (95% CI: 0.72–0.96; *p* < 0.01) (Figure 5b). In the whole cohort, it was 0.84 (95% CI: 0.74–0.94; *p* < 0.01) (Figure 5c). When the cutoff value was set to ≥8 mm^2^, the cutoff value's sensitivity was 92.5%.

## DISCUSSION

The results of whitish core amount calculated by the AMUS strongly correlated with SVWC assessments performed manually. It demonstrated high sensitivity for the histological diagnosis using a cutoff value of ≥8 mm^2^. We recommend the AMUS as it is simpler than the previously reported SIPS for EUS‐FNAB diagnosis.^4^

Several previous studies indicated that ROSE significantly improved the diagnostic accuracy of EUS‐FNAB.^9^, ^10^, ^11^, ^12^ However, ROSE is not feasible in all institutions where EUS‐FNAB is performed owing to a lack of financial and other hospital resources and a shortage of available cytopathologists. Furthermore, recent randomized control trials and meta‐analyses have questioned the effectiveness of ROSE.^13^, ^14^, ^15^ Bang et al^16^ concluded that the advanced diagnostic capabilities of new‐generation biopsy needles might eliminate the need for ROSE. However, in centers with low adequacy rates (<90%) and less experienced endoscopists, ROSE may play a significant role.^17^ Iwashita et al^18^ reported that macroscopic on‐site quality evaluation (MOSE) using 19‐gauge needles was a simpler alternative to ROSE and provided an essential index for performing EUS‐FNAB in hospitals unequipped for ROSE. Furthermore, although recent studies have reported that MOSE is useful even when using a 22‐gauge needle,^19^, ^20^, ^21^, ^22^ determining the presence or absence of whitish core could be difficult for samples with significant blood contamination. We believe SIPS, which is performed under a stereomicroscope, provides a more accurate determination of the presence or absence of whitish core and its amount (length). We have reported that the sensitivity for histological diagnosis was high (91.4%) when the SVWC cutoff value was ≥11 mm.^4^ However, in two previously reported prospective studies on SIPS,^4^, ^5^ SVWC and red component isolation were evaluated by only two specific physicians. Thus, to generalize the application of SIPS, an objective evaluation method is required. Although manual evaluation of SVWC can be performed quickly, the steps for isolating SVWC and red components are complicated and prolong sample preparation time. Therefore, we developed an AMUS that can be introduced at any facility and provides objective assessments. We verified the correlation between manual SVWC assessments and the AMUS and the isolation effect in SIPS (differences in histological adequacy scoring depending on whether there is isolation) for the first time through subgroup analysis. The AMUS demonstrated a high correlation with manual SVWC assessment. Additionally, we were able to verify that a high diagnostic sensitivity could be achieved with the AMUS when the cutoff value was ≥8 mm^2^.

The findings following subgroup analysis indicated that by isolating SVWC and red components, which is a process of SIPS, the degree of blood component contamination in the sample reduced significantly; however, there were no differences in the adequacy score or tumor cell content. The reasons for this are as follows: first, the red component, although minimal, may have contained cancer tissues; second, even if tumor tissues can be densely arranged by isolation, as the interstitium is abundant in PC, it would not be sufficient to increase the tumor cell content. However, as only 117 samples were obtained from 30 subjects in this study, definite conclusions cannot be made. Therefore, a separate prospective study should be performed to verify whether similar results can be obtained from the SIPS process even without isolation.

Genome analysis using FFPE for treatment selection in patients with PC is becoming increasingly prevalent and is expected to increase even further. Thus, high‐quality samples should be prepared with higher tumor cell content. The criterion for analysis in FoundationOne (Foundation Medicine Inc, Cambridge, MA, USA), involving one of the next‐generation sequencing technologies, is that over 20% of the total nucleated cells should be tumor cells.^23^ Overall, the tumor cell content was ≥20% in 53 of 74 samples (71.6%) with SVWC ≥ 11 mm, an indicator for histological diagnosis. Furthermore, the tumor cell content was ≥20% in 25 of 31 samples (80.6%) with SVWC ≥ 22 mm, twice the cutoff value for histological diagnosis. In 117 AMUS‐evaluated specimens, the tumor cell content was ≥20% in 56 of 80 samples (70%); the cutoff value of the whitish core was ≥8 mm^2^. In future, these may serve as new reference values for estimating whether the tumor cell content required for genome analysis has been obtained.

Despite these favorable results, several limitations of the AMUS need to be addressed. First, the process has not been fully automated and requires human intervention. To avoid sample overlapping, stringy samples taken by EUS‐FNAB must be organized manually onto a petri dish and soaked in formalin solution. If the SVWC is located below overlapping samples, it may not be detected and may be underestimated during multiband image acquisition by the automatic analyzer. Second, the inability to perform ROSE is usually attributed to the lack of an on‐site cytopathologist. Contrarily, the AMUS requires only adequate equipment preparation. Because it is still in the development stage, we cannot estimate the costs of using the AMUS.

Given the limitations of this study's exploratory nature, a power analysis could not be performed; therefore, an achievable target of 30 patients (120 specimens) was selected. Thus, it is necessary to conduct a prospective study involving multiple centers and more cases. The primary endpoint should be the achievement of similar high diagnostic sensitivity with a cutoff value ≥ 8 mm^2^.

In conclusion, the outcomes of the AMUS are similar to those of SIPS. The AMUS can be used as an alternative to SIPS, which requires proficiency and involves time‐consuming procedures. Our findings may provide useful new indices for EUS‐FNAB, particularly in institutions where ROSE cannot be performed. Further multicenter studies are required to validate our findings.

## CONFLICT OF INTEREST

The authors declare that they have no conflict of interest.

## FUNDING INFORMATION

A research grant from Olympus Corporation supported this study.
